# A Fine Differentiation: Chlorpyrifos and Neuronal Development

**Published:** 2006-05

**Authors:** Julian Josephson

Although chlorpyrifos has been restricted for use in the home, it is still permitted for agriculture and remains the most widely used organophosphate pesticide in the world. Animal studies and *in vitro* models have indicated that chlorpyrifos has direct and indirect effects on fetal and neonatal neural cell replication and differentiation. These effects include cholinesterase inhibition as well as immediate and delayed-onset changes in synapse formation, neurotransmitter release, neurotransmitter receptor expression, and intracellular signaling. Moreover, chlorpyrifos can exert simultaneous, opposite effects on axonal and dendritic growth. Now researchers from Duke University Medical Center show that chlorpyrifos has direct effects on the differentiation that determines the phenotypic fate of developing neuronotypic cells **[*EHP* 114:667–672; Jameson et al.]**.

One problem with *in vivo* animal studies is the difficulty of teasing out the indirect effects mediated by mother–fetus or mother–neonate interactions, as opposed to the direct effects of chlorpyrifos on the developing brain. Accordingly, attention has increasingly come to focus on *in vitro* models that simulate the development of two basic types of brain cells, neurons and glia.

The Duke researchers set up such a model using PC12 cells, a tumor cell line that originates from a neuronal phenotype and that can recapitulate all the major phases of neurodevelopment thought to be targets for chlorpyrifos. With the addition of the peptide known as nerve growth factor, differentiation begins: PC12 cells cease dividing and develop the characteristics of neurons, including axonal projections and specialization into either cholinergic or catecholaminergic transmitter systems.

Cholinergic systems have shown immediate and lasting damage when exposure to chlorpyrifos occurs during periods of rapid cell replication (when the neuronal cells are dividing) and differentiation. In contrast, chlorpyrifos exposure initially enhances the development of catecholaminergic systems, increasing the expression of the proteins characteristic of this system and enhancing synaptic activity; nevertheless, long-term brain function deficits eventually appear, mainly in the form of disruption of synaptic connectivity. The current study was aimed at answering three basic questions about *in vitro* exposure to chlorpyrifos: Does chlorpyrifos alter the ability of developing neurons to express a specific neurotransmitter phenotype? If so, at what stage of cell maturation does this occur? And do such changes occur at chlorpyrifos concentrations below those that affect cell viability?

The researchers evaluated PC12 cells in the undifferentiated state, at the initiation of differentiation, and at mid-differentiation. They contrasted the effects on cell viability, DNA synthesis associated with cell replication, and increased expression of enzyme markers that characterize cholinergic or catecholaminergic phenotypes: choline acetyltranferase (ChAT) and tyrosine hydroxylase (TH), respectively.

Chlorpyrifos exposure at the start of differentiation significantly reduced ChAT but not TH activity. With chlorpyrifos addition during mid-differentiation (four days after nerve growth factor pretreatment), ChAT was unaffected, but TH was increased slightly. Chlorpyrifos reduced DNA synthesis in the undifferentiated state, thereby impairing general neuronal cell development, whereas at the start of differentiation, it specifically impeded development of the cholinergic phenotype.

Chlorpyrifos administration *in vivo* is known to cause deficits in the number of neurons and cholinergic function. Because the researchers were able to reproduce these effects reliably *in vitro*, they suggest that chlorpyrifos directly influences the phenotypic fate of neuronal precursors. In addition, they suggest that their cell culture model could become useful for the rapid screening of neurodevelopmental outcomes with related, or even disparate, neurotoxicants.

